# R353Q polymorphism in the factor VII gene and cardiovascular risk in Heterozygous Familial Hypercholesterolemia: a case-control study

**DOI:** 10.1186/1476-511X-10-50

**Published:** 2011-04-09

**Authors:** Juan Criado-García, Francisco Fuentes, Cristina Cruz-Teno, Antonio García-Rios, Anabel Jiménez-Morales, Javier Delgado-Lista, Pedro Mata, Rodrigo Alonso, José López-Miranda, Francisco Pérez-Jiménez

**Affiliations:** 1 Lipids and Atherosclerosis Unit, Department of Medicine, IMIBIC/Hospital Universitario Reina Sofía/Universidad de Córdoba, Córdoba, Spain and CIBER Fisiopatología Obesidad y Nutrición (CIBEROBN), Instituto de Salud Carlos III, Madrid, Spain; 2 Lipid Clinic. Internal Medicine. IIS- Fundación Jiménez Díaz, Madrid, Spain

**Keywords:** Familial Hypercholesterolemia, Factor VII, R353Q polymorphism

## Abstract

**Background:**

Heterozygous Familial Hypercholesterolemia (FH) is a genetic disorder characterized by a high risk of cardiovascular disease. Certain polymorphisms of the factor VII gene have been associated with the development of coronary artery disease and there is a known association between factor VII levels and polymorphic variants in this gene. To date, no study has evaluated the association between factor VII and coronary artery disease in patients with FH.

**Results:**

This case-control study comprised 720 patients (546 with FH and 174 controls). We determined the prevalence and allele frequencies of the R353Q polymorphism of factor VII, the plasma levels of factor VII antigen (FVII Ag) and whether they could be predictive factors for cardiovascular risk. 75% (410) of the patients with FH were RR, 23% (127) RQ and 1.6% (9) QQ; in the control group 75.3% (131) were RR, 21.3% (37) RQ and 3.4% (6) QQ (p = 0.32). No statistically significant associations were observed in the distribution of genotypes and allele frequencies between case (FH) and control groups. Nor did we find differences when we evaluated the relationship between the R353Q polymorphism and cardiovascular risk (including coronary disease, ischemic stroke and peripheral arterial disease), either in the univariate analysis or after adjustment for sex, age, arterial hypertension, body mass index, xanthomas, diabetes, smoking, HDLc and LDLc and lipid-lowering treatment. The FVII Ag concentrations behaved in a similar fashion, with no differences for the interaction between controls and those with FH (RR vs. RQ/QQ; p = 0.96). In the subgroup of patients with FH no association was found among cardiovascular disease, genotype and FVII Ag levels (RR vs. RQ/QQ; p = 0.97).

**Conclusions:**

Our study did not find a direct relationship between cardiovascular risk in patients with Heterozygous Familial Hypercholesterolemia, the R353Q polymorphism of factor VII and FVII Ag levels.

## Bakground

Heterozygous Familial Hypercholesterolemia (FH) is an autosomal codominant disease caused by defects in the low-density lipoprotein receptor (LDLR) gene [1]. It is a genetic disorder characterized by elevated levels of plasma low-density lipoproteins cholesterol (LDLc) ranging from 300 to 400 mg/dl, corneal arcus, tendon xanthomas and a high prevalence of early-onset coronary disease. It is extremely heterogeneous, with an incidence of one in 500 persons (0.2%) [2,3]. The study of the mechanisms that encourage the development of cardiovascular disease (CVD) is of great interest, given the variability both of the clinical picture and in the therapeutic response that characterizes this disease. Moreover, not all diagnosed patients present clinical symptoms, so a better understanding of the factors that lead to some patients developing early-onset CVD while others do not, would be very useful as a means of evaluating the risk and adopting preventive measures.

In patients at high CVD risk, such as patients with FH, there appears to be a chronic activation of the mechanisms of coagulation and hemostasis, which leads to what is regarded as a permanent prothrombotic condition [4,5]. Thrombosis is the basis of the most acute manifestations of coronary artery disease. The atheromatous plaque rupture, with the consequent exposure of tissue factor to blood and its subsequent union with factor VII (FVII), initiates the coagulation cascade [6]. One of the components of hemostasis that has aroused interest for its potential role in the development of coronary disease and stroke is FVII. Some studies have associated high levels of plasma FVII with an elevated risk of coronary artery disease [7-10], although these findings have not been confirmed by other studies leading to highly variable results [11,12]. The R353Q polymorphism of the FVII gene has been among those most closely associated with variations in plasma levels of FVII [13-16]. This is a simple nucleotide polymorphism (SNP), characterized by the substitution of a guanine base by an adenine, which involves the substitution of arginine (R) by glutamine (Q) in codon 353 of this protein [8]. Lower levels of FVII have been detected in carriers of the Q allele than in individuals who are homozygous for the more common R allele [17,18]. Carriers of the Q allele may therefore be protected against acute thrombotic events, as has been demonstrated by a number of case-control studies in which the Q allele was associated with a reduced risk of acute myocardial infarct [19-24]; however, this association was weaker [25] or was not detected in another studies [26-28].

The principal objectives of this study were therefore to analyze the prevalence and allele frequency of the R353Q polymorphism and the plasma FVII levels in FH patients with or without CVD and in family members who were unaffected by FH (controls), and to determine whether the presence of this polymorphism could be a predictor of CVD risk in these patients.

## Material and methods

### Study design and study population

This was a case-control study of a sample population selected from a Spanish FH Longitudinal Cohort Study, supported by the "Fundación Española de Hipercolesterolemia Familiar" *http://www.colesterolfamiliar.com*. One person per familiy (index case) was also included. The clinical diagnosis of FH was made in accordance with the diagnostic criteria homogenized by the MEDPED international cooperative program, which is coordinated by the World Health Organization (WHO) [29]. All patients with FH included in the study were heterozygous carriers for known functional mutations in the LDLR gene. This FH genetic diagnosis was carried out also in control patients, assuring the no-FH diagnosis. A written informed consent was obtained from all participants before their inclusion in the cohort and the protocol was approved by the ethic committee of the CEIC Fundación Jiménez Díaz (Madrid). STREGA criteria were used in the reporting of our study [30,31].

We determined the polymorphism in 720 patients. Among them there were 546 persons affected by FH (cases) and 174 family members unaffected by FH (controls). Clinical data concerning sex, age, history of arterial hypertension (HT), smoking, diabetes mellitus (DM), body mass index (BMI), xanthomas, total cholesterol level, triglycerides, LDLc, high-density lipoproteins cholesterol (HDLc), treatment for hyperlipemia and CVD were collected.

### Cardiovascular events

The evaluation and definition of CVD events was based on the WHO MONICA project [32]. CVD was classified as "early onset" when it occurred at an age of less than 55 years in men and 65 in women. CVD events were evaluated via analysis of the CVD history of the patient himself and those of his first-degree (parents, siblings and children) and second-degree (grandparents, aunts and uncles, cousins and nieces and nephews by blood) family who had a documented clinical history of a) ischemic cardiopulmonary (myocardial infarct, angina pectoris, surgery or any other coronary revascularization procedure), b) cerebrovascular disease or c) peripheral vascular disease.

### Sampling procedures

On enrollment in the study, blood samples were obtained by venous puncture from fasting patients. Samples were sent to a central laboratory for the extraction of genomic DNA from the leukocyte fraction of fresh blood, using the Puregen^® ^(DNA isolation kit, Gentra Systems, MN, U.S.A). The genomic DNA was used for the determination of the R353Q mutation of the FVII coagulation gene.

### Determination of FVII polymorphism

FVII polymorphism was determined according to Lindman et al. [33], by real-time polymerase chain reaction (RT-PCR) (Stratagene Mx3005P Cultek) of a DNA region of exon 8 of the FVII coagulation gene. We used 150 ng of genomic DNA, 3 mM of MgCl2, 200 μM of each nucleotide, 1 IU of Hot Start polymerase and reaction buffer to 1X (Dominion-MBL).

The primers were mixed with the probe (GATGCCCGTCAGGTACCACGTGCCC (C/T) GGTAGTGGGTGGCATGTGGGCCTCC) to 1X in a final volume of 10 μl (Taqman^®^-Applied Biosystems). The DNA was denatured at 95°C for 5 min, followed by 40 cycles of denaturation at 95°C for 30 s, a phase of primer-polymerase union (60°C for 1 min) and extension (72°C for 30 s). The population genotypes were subsequently analyzed using the curves supplied by the RT-PRC equipment. In the R353Q assay, a guanine base is replaced by an adenine, which requires the substitution of arginine (R) by glutamine (Q) in codon 353 of this protein.

Standard good laboratory practices were undertaken to ensure the accuracy of genotype data, including the inclusion of dummy duplicates.

### Determination of plasma FVII Ag concentrations

FVII Ag levels were determined using an Elisa Kit (AssayMax Human Factor VII ELISA Kit^®^, Assaypro) with a minimum detectable dose < 6 ng/ml and an intra and inter-assay coefficients of variation of 5.0 % and 7.1% respectively.

### Quantification of plasma lipid levels

Plasma lipid levels (mg/dl) were determined by spectrophotometry (enzymetric colorimetry) in a Modular Analytics ISE-4-DDPPEEPP autoanalyzer (F. Hoffmann-La Roche, Basle, Switzerland); an oxidation-peroxidation method was employed to assay total cholesterol, HDLc and triglycerides; LDLc was estimated by Friedewald's formula (LDLc = total cholesterol - triglycerides/5-HDLc).

### Statistical analysis

The SPSS statistical package (version 17.0 for Windows) was utilized for all statistical analyses, which comprised:

- descriptive analysis of absolute and relative frequencies with 95% confidence intervals (CI) and means and standard deviations of the quantitative variables.

- comparison of qualitative variables using chi-squared contingency tables; in the case of 2 × 2 tables, the chi-squared table alone was used, while if any expected frequency was < 5, Fisher's exact test was employed.

- comparisons of the mean values of the quantitative variables by means of Student's t-test for two independent samples or single-factor analysis of variance for more than two independent samples.

In all the statistical tests, values of p < 0.05 were treated as significant and the hypothesized contrasts were bilateral.

In order to evaluate the association of the FVII genotype with CVD, we calculated the odds ratio (crude OR) and its 95% CI by univariate logistical regressions. In order to control for sex, age, HT, BMI, xanthomas, DM, LDLc, HDLc, smoking and lipid-lowering therapy, we employed a multivariate logistics model (adjusted OR).

## Results

### Baseline characteristics

The general characteristics of the patients are shown in Table 1. No significant differences were found in the following variables: sex, BMI, HT, DM or triglyceride levels between the FH and control groups. However there were differences in age, total cholesterol, LDLc and HDLc levels, xanthomas (102 of 546 HF patients developed them, 18.9%), in those patients under lipid-lowering treatment (more than 83% of the FH), in the number of CVD events as well as in global CVD and in the mean age of the first event.

**Table 1:** Basic characteristics of patients and controls

	Controls *N = 174*	Familial hypercholesterolemia *N = 546*	Value of P
Age (years)	39.7 ± 17.5	43.5 ± 16.3	**0.009**

Women (%)	55.7	47.3	0.62

BMI	25.7	26.6	0.20

HT (%)	10.3	12.7	0.41

DM (%)	2.3	2.4	1.000

Total cholesterol (mg/dl)	239.6 ± 70.3	270.3 ± 70.4	**< 0.001**

Triglycerides (mg/dl)	102.2 ± 61.1	109.1 ± 62.5	0.22

LDLc (mg/dl)	168.4 ± 68.4	201.3 ± 67.1	**< 0.001**

HDLc (mg/dl)	50.2 ± 13.4	46.1 ± 13.2	**< 0.001**

Xanthomas	0.0	102 (18.6%)	**< 0.001**

Smoking (%)	46.8	49.4	0.55

Lipid-lowering treatment	66 (37.9%)	458 (83.9%)	**< 0.001**

CV disease	8 (4.6%)	80 (14.7%)	**< 0.001**

First CV event (years)	57.1 ± 13.3	46.7 ± 11.1	**0.01**

Coronary artery disease	6 (3.4%)	75 (13.7%)	**< 0.001**

Cerebrovascular disease	3 (1.7%)	4 (0.7%)	0.37

Peripheral arterial disease	1 (0.5%)	12 (2.1%)	0.21

CVD distribution:			
-single territory	6 (3.5%)	71 (13%)	**< 0.001**
-two or more territories	2 (1.2%)	9 (1.6%)	

Values expressed as means ± standard deviation. **BMI: **body mass index, calculated by dividing weight by height squared. **HT: **arterial hypertension. **DM: **diabetes mellitus. **CV: **cardiovascular. **LDLc: **low-density lipoproteins cholesterol. **HDLc: **high-density lipoproteins cholesterol.

### Cardiovascular disease

In 14.7% of the patients with FH a CVD event occurred; 75 patients developed coronary artery disease, 4 cerebrovascular disease and 12 peripheral arterial disease. In the distribution by vascular territories, 71% of FH with CVD had affectation of a single territory while in 9%, two or more territories were affected, with a statistically significant difference in the distribution of the CVD events compared with the control group (p < 0.001). We must emphasize the low frequency of cerebrovascular disease in both the control (1.7%) and the FH group (0.7%), with no significant difference between them (p = 0.37). The frequency of peripheral vascular disease (predominantly in the lower extremities) was 2.1% in FH patients and 0.5% in the controls (p = 0.21).

### The R353Q polymorphism

In the analysis of the R353Q genotypes distribution (Table 2), we found that 75.1% of the patients with FH were RR, 21.3% were RQ and 3.4% QQ, without any evidence of differences between the control and FH groups (p = 0.32). Similar results were obtained when we analyzed this genotypes distribution comparing RR vs RQ/QQ (p = 0.96). In the allele frequencies (R or Q allele presence), we found no statistically significant differences between case (FH) and control groups (0.86 and 0.87, IC 95% 0.82, 0.89 and 0.85, 0.89 respectively for the R allele; 0.14 and 0.13, IC 95% 0.11, 0.18 and 0.11, 0.15 respectively for the Q allele). The allele frequencies in both were adjusted to the Hardy-Weinberg equilibrium (p = 0.97 and p = 28, respectively).

**Table 2:** FVII genotype polymorphisms of FH and control patients

	Controls *N = 174*	Familiar hypercholesterolemia *N = 546*	Total *N = 720*	Value of P*
Genotype R353Q FVII				0.32

*RR*	131 (75.3%)	410 (75.1%)	541 (75.1%)	

*RQ*	37 (21.3%)	127 (23.3%)	164 (22.8%)	

*QQ*	6 (3.4%)	9 (1.6%)	15 (2.1%)	

				0.96

*RR*	131 (75.3%)	410 (75.1%)	541 (75.1%)	

*RQ/QQ*	43 (24.7%)	136 (24.9%)	179 (24.9%)	

Allele frequency				
*R*	0.86 (0.82-0.89)	0.87 (0.85-0.89)		
*Q*	0.14 (0.11-0.18)	0.13 (0.11-0.15)		

*P values expressed by χ2 (Pearson). **FVII**: Factor VII.

### Polymorphism and cardiovascular risk

In order to identify possible relationships between the different genotypes and higher or lower prevalence of CVD in FH patients (Table 3), we performed an univariate analysis. With respect to global CVD we found no statistical differences between carriers of the RR and RQ genotypes, just as in those subjects who were homozygous for the non-dominant gene (RR genotype). The separately analysis of coronary artery disease and peripheral vascular disease led to same results; this analysis could not be applied to cerebrovascular disease because of its low incidence (four subjects). Multivariate logistic regression analysis, adjusted for sex, age, HT, BMI, xanthomas, DM, HDLc, LDLc, smoking habit and treatment for hyperlipemia found no association between the genotypes of the FVII R353Q mutation and CVD.

**Table 3:** Multivariate analysis of FH patients

		CVD Patients (%)					
	**Patients** **(n = 546)**	**No** **(n = 466)**	**Yes** **(n = 80)**	OR (CI 95%)*	**P**	Adjusted OR** (CI 95%)	**P**

**FVII**							
RR	410	350 (75.1)	60 (75.0)	1.00 (Reference)		1.00 (Reference)	
RQ	127	110 (23.6)	17 (21.3)	0.89 (0.50 - 1.59)	0.70	0.76 (0.36 - 1.58)	0.46
QQ	9	6 (1.3)	3 (3.7)	2.88 (0.70 -11.84)	0.14	3.53 (0.66 - 18.92)	0.14
RQ/QQ	136	116 (24.9)	20 (25.0)	0.99 (0.58 - 1.72)	0.98	0.89 (0.45 - 1.78)	0.75

		**CAD Patients (%)**					

	**Patients** **(n = 546)**	**No** **(n = 471)**	**Yes** **(n = 75)**	OR (CI 95%)*	**P**	Adjusted OR** (CI 95%)	**P**

**FVII**							
RR	410	354 (75.1)	56 (74.7)	1.00 (Reference)		1.00 (Reference)	
RQ	127	111 (23.5)	16 (21.3)	0.90 (0.50 - 1.63)	0.73	0.90 (0.43 - 1.87)	0.77
QQ	9	6 (1.4)	3 (4.0)	3.12 (0.76 -12.86)	0.11	3.33 (0.64 - 17.24)	0.15
RQ/QQ	136	117 (24.9)	19 (24.3)	1.02 (0.58 - 1.78)	0.96	1.05 (0.52 - 2.08)	0.90

		**PAD Patients (%)**					

	**Patients** **(n = 546)**	**No** **(n = 534)**	**Yes** **(n = 12)**	OR (CI 95%)*	**P**	Adjusted OR** (CI 95%)	**P**

**FVII**							
RR	410	401 (75.1)	9 (75.0)	1.00 (Reference)		1.00 (Reference)	
RQ	127	125 (23.4)	2 (13.7)	0.71 (0.15 -3.34)	0.67	0.78 (0.15 - 3.84)	0.76
QQ	9	8 (1.5)	1 (8.3)	5.56 (0.63 -49.33)	0.12	5.77 (0.26 - 125.96)	0.26
RQ/QQ	136	132 (24.9)	3 (24.0)	1.00 (0.27 - 3.77)	0.99	1.03 (0.26 - 4.17)	0.96

*Crude OR obtained by univariate logistic regression analysis. **Multivariate logistic regression adjusted for sex, age, body mass index, xanthomas, arterial hypertension, smoking, diabetes mellitus, LDLc, HDLc and lipid-lowering treatment. **FVII: **Factor VII. **FH**: Heterozygous Familial Hypercholesterolemia. **CVD: **cardiovascular disease. **CAD: **coronary artery disease. **PAD: **peripheral artery disease.

### FVII Ag levels

Plasma FVII Ag levels (ng/mL) were determined in a subgroup of 320 patients, 160 controls (including 8 with CVD) and 160 FH patients (80 with CVD). The genotypes distribution (RR vs RQ/QQ) between both groups was similar (p > 0.05). Analysis of variance for two factors (FH and genotype) adjusted for sex, age, DM, smoking, HT and CVD was applied and no differences were found in FVII Ag levels for the interaction between both factors (p = 0.96) (Table 4). We also studied the FVII Ag concentration in patients with FH (n = 160) depending on the presence or not of CVD; in the analysis of variance for CVD and genotype adjusted for age, sex, DM, smoking and HT no differences were found for the interaction between them (p = 0.97) (Table 5).

**Table 4:** FVII Ag levels in controls and FH patients

		Controls (n = 160)	Familiar hypercholesterolemia (n = 160)
**Genotype** **R353Q FVII**	**FVII levels** **(ng/mL)**		

RR *(n = 120)*	Mean	374.6 ± 239.6	292.8 ± 157.6
	
	Adjusted mean	372.9 ± 204.7	288.6 ± 211.2

RQ/QQ *(n = 40)*	Mean	387.5 ± 248.2	265.6 ± 130.8
	
	Adjusted mean	376.7 ± 198.1	293.9 ± 200.2

Values expressed as means ± standard deviation. Analysis of variance for two factors (FH and genotype) adjusted for age, sex, diabetes, smoking, arterial hypertension and cardiovascular disease. No differences were found for the interaction between both factors (0.96). **FVII: **Factor VII.

**Table 5:** FVII Ag levels in FH patients and CVD

		CVD	
		
		No (n = 80)	Yes (n = 80)
**Genotype** **R353Q FVII**	**FVII levels** **(ng/mL)**		

RR *(n = 60)*	Mean	307.1 ± 175.4	278.6 ± 137.5
	
	Adjusted mean	260.9 ± 240.0	311.7 ± 167.7

RQ/QQ *(n = 20)*	Mean	219.7 ± 111.2	311.4 ± 135.5
	
	Adjusted mean	213.3 ± 168.1	359.9 ± 152.3

Values expressed as means ± standard deviation. Analysis of variance for two factors (CVD and genotype) adjusted for age, sex, diabetes mellitus, smoking and arterial hypertension. No differences were found for the interaction between both factors (0.97). **CVD: **cardiovascular disease. **FVII: **Factor VII.

## Discussion

In our study, the R353Q polymorphism of the FVII gene, its allele frequency and the FVII Ag levels neither predict nor are directly related to the prevalence of CVD in FH patients.

FH is the monogenetic disease with the highest prevalence in human beings and it is associated with a high risk of early and serious CVD. The global prevalence of CVD in this study was 14.7%, with a mean age of 45.5 ± 16.3 years, while it is estimated that in general spanish population the prevalence in the same age range is 2.6% [34]. The prevalence of FH is highly variable in western populations, being as high as 39% in some series [35]. These variations have been attributed to a number of factors, such as the different diagnostic criteria (genetic or clinical) used in the FH studies, differences in the methodologies employed and the possible direct influence of environmental factors, such as diet. In fact, it has been demonstrated that consumption of a Mediterranean diet rich in olive oil reduces the incidence of CVD in the general population [34], even leading to a reduction in the level of activated FVII [37], which would also have a direct influence on subjects with FH. The phenotypic expression of FH in terms of onset and severity of atherosclerotic vascular disease varies considerably. A paucity of consistent data exist on factors that contribute to these phenotypic differences. Several studies have analyzed the influence of traditional CVD risk factors and the functional variety of LDLR mutation on this phenotypic variability [38,39]. However, they can only partially explain the observed differences. Therefore other still unknown factors, such as genetic conditions, could play an important role in the development of CVD in these patients. This is sustained by the fact that clustering of CVD occurs in FH kindred. The genetic variability of this population sample would be determined by the presence or absence of certain polymorphisms as potential predictor of CVD risk. Also a number of studies have demonstrated that FH patients with xanthomas have a higher risk of CVD compared to those without them [40,41]. A recent meta-analysis has demonstrated this fact concluding that the presence of tendon xanthomas is associated with a 3 times higher risk of CVD among FH patients, suggesting that xanthomas and CVD may share a common etiology [42]. Our population had a 14.7% of global CVD prevalence, very low if we compare it with another FH cohort as Simon Broome [43], whose global prevalence was much higher (60%). In Simon Broome cohort, 48% of FH patients presented with tendon xanthomas; this fact could partially justify the highest prevalence of CVD when we compared it with our study where only 18.6% developed them. This low prevalence of CVD could be explained by the low presence of tendon xanthomas in our population. On the other hand we should also considered that Simon Broome cohort used clinical criteria for FH diagnosis; in our cohort genetic diagnosis of FH was carried out in all cases.

Among those potentially interesting genes of such phenotypic variability is FVII gene. Several of its polymorphisms have been associated with differences in the risk of suffering CVD events when they have been analyzed in groups of severe atherosclerosis patients [18,19,23,24], basically due to variations in their levels and plasma activity, which may encourage a state of hypercoagulability [13,16]. Our study analyzed the different types of the R353Q polymorphism of FVII gene in a sample of FH patients, comparing their potential association with CVD (coronary, cerebrovascular and peripheral artery disease). Some studies have suggested that global CVD risk appears to be independent of the R353Q polymorphism of FVII [44-46]; however, a meta-analysis has demonstrated that bearers of the Q allele are at lesser risk [47]. Furthermore, a lower rate of myocardial infarcts has been observed in such populations [23,24]. Several studies have described a reduction in circulating FVII levels in patients who are heterozygous (RQ) or homozygous (QQ) for the non-dominant gene thus associating these levels with the presence of the Q allele [13,14]. One study by Girelli et al. has even suggested a possible protective effect of this allele, as a means of explaining why patients with severe coronary arteriosclerosis do not evolve acute myocardial infarct [18]. In the face of these results, the data from our study, in which the frequency distribution of the various FVII alleles is identical to that of the general population [33,48], do not shown any differences in terms of CVD frequency between the subjects who are homozygous for the various alleles when patients, with or without FH, are compared. Although FH patients are at high risk of CVD, they do not present differences in the frequency of CVD events as a function of being carriers, or not, of either allele. The fact that these patients present elevated levels of total cholesterol and LDLc, and lower levels of HDLc (these being factors that are independent of the development of early-onset cardiovascular phenomena), may have influenced our results, without such findings having been observed in association with allele Q. On the other hand, there was no causal relationship between the carriers of the R allele, which determines the most frequent genotype in the population, and development of CVD.

Respecting plasma FVII Ag levels our data do not shown differences in controls and FH patients and no relation could be established with the genotype and also with its allelic distribution (RR vs. RQ/QQ). These results contrast with some studies that found lower FVII levels in carriers of Q allele [13] and moreover no protective effect could be attributed to Q allele in our population [14]. In the subgroup of patients affected of FH, the comparison between those with or without CVD showed no change in the adjusted mean levels of FVII depending on genotype. All these circumstances would support the absence of association between the R353Q polymorphism and CVD risk in our FH patients, according to all those studies where global CVD risk appears to be independent of the R353Q polymorphism of FVII [44-46].

We must also consider, as one of the possible reasons that could justify this lack of relation between the polymorphism and CVD, the low number of cardiovascular events developed in our FH patients (14.7%), probably because of their mean age, just 43.5 ± 16.3 with an age in the first CV event of 46.7 ± 11.1 years. Jansen et al [49], who investigated the contribution of polymorphisms in multiple candidate genes to CVD risk, found a 33.1% of CVD frequency in their FH cohort, where the mean age of onset of CVD was 48.2 years, although they analyzed patients with age at last visit of 56.4 ± 11.4 years.

## Conclusions

We are unaware of any study that has associated FVII with CVD risk in FH patients and our study is thus a pioneer in this respect. It has been well demonstrated that there are variations in the concentration and activity of FVII as a function of the genotype involved, with these being lower in carriers of the Q allele. As our data have shown, therefore, the R353Q polymorphism of FVII gene does not predict cardiovascular risk in the sample of FH patients in the Spanish Cohort.

## List of Abbreviations

FH: Heterozygous Familial Hypercholesterolemia; LDLR: low-density lipoprotein receptor; CVD: cardiovascular disease; FVII: factor VII; FVII Ag: factor VII antigen; HDLc: high-density lipoproteins cholesterol; LDLc: low-density lipoproteins cholesterol; DM: diabetes mellitus; BMI: body mass index; HT: arterial hypertension.

## Disclosures

None of the authors had any conflict of interest.

## Authors' contributions

JCG: Development of study design, determination of FVII polymorphism and FVII Ag concentrations, statistical analysis and interpretation, draft manuscript. FF: Development of study design, collection data and analysis and interpretation. CCT: Determination of FVII polymorphism and FVII Ag concentrations. AGR: Collection data. AJM: Development of study design, statistical analysis. JDL: Statistical analysis. PM: Collection and data interpretation. RA: Collection and data interpretation. JLM: Development of study design, statistical analysis and interpretation. FPJ: Development of study design, statistical analysis and interpretation. All authors have read and approved the final manuscript.

## Funding Sources

This work was supported by research grants from the Centro Nacional de Investigaciones Cardiovasculares (CNIC-08-2008), National Health Institute; CIBER (CBO/6/03), Instituto de Salud Carlos III; CICYT (SAF 01/2466-C05 04 to F P-J, SAF 01/0366 to J L-M, AGL 2004-07907 to J L-M, AGL 2006-01979 to JL-M), the Spanish Ministry of Health (FIS 01/0449, FIS PI041619 to CM); Fundación Cultural "Hospital Reina Sofía-Cajasur; Consejería de Salud, Servicio Andaluz de Salud (00/212, 00/39, 01/239, 01/243, 02/64, 02/65, 02/78, 03/73, 03/75, 04/237, 04/191, 04/238, 05/396); Consejería de Educación, Plan Andaluz de Investigación, Universidad de Córdoba; Centro Excelencia Investigadora Aceite de Oliva y Salud (CEAS); NIH grants HL54776 and DK07503; Fundación Española de Hipercolesterolemia Familiar.
